# Pediatric and School-Age Vision Screening in the United States: Rationale, Components, and Future Directions

**DOI:** 10.3390/children10030490

**Published:** 2023-03-02

**Authors:** Christina Ambrosino, Xi Dai, Bani Antonio Aguirre, Megan E. Collins

**Affiliations:** 1 Johns Hopkins School of Medicine, Baltimore, MD 21205, USA; 2 Wilmer Eye Institute, Johns Hopkins School of Medicine, Baltimore, MD 21287, USA; 3 Berman Institute of Bioethics, Johns Hopkins University, Baltimore, MD 21205, USA

**Keywords:** vision screening, refractive error, amblyopia, school-based vision programs

## Abstract

Pediatric vision screening detects children at risk for visual conditions with the goal of connecting those in need with an eye care provider for evaluation and treatment. The primary aim for vision screening in younger children is the detection of those at risk for amblyopia, which can result in irreversible vision loss if left untreated. In older children, screening goals broaden to include the detection of risk for uncorrected refractive error. In the United States, professional organization guidelines and state-mandated requirements for vision screening vary widely across both the timing and components of screening. In this article, we describe the goals and components of pediatric vision screenings, current challenges, novel approaches to providing follow-up services through school-based vision programs, and future directions.

## 1. Introduction

Visual function is an integral part of a child’s overall health and quality of life and plays an important role in children’s physical, intellectual, social, and emotional development. Vision has implications in the educational setting, where better vision has been associated with improved academic performance [1]. Further, educational achievement, an important social determinant of health, bears a lifetime of benefits for all aspects of health, including vision health [2]. This inter-relatedness of health and education underlies the Whole School, Whole Community, Whole Child (WSCC) model’s approach to child health and well-being [3]. This WSCC approach necessitates multidisciplinary coordination among primary pediatric providers, eye care professionals, teachers, and school health teams to ensure vision problems in children are addressed in a timely manner. In this article, we describe the goals and components of pediatric vision screenings in the pre-school and school-age population, current challenges, novel approaches to providing follow-up services through school-based vision programs, and future directions.

In Part 1 of this document, we aim to provide a brief overview of pre-school and school-aged vision screening guidelines in the United States and common eye conditions that vision screenings target. In addition, we provide a discussion of opportunities to coordinate vision screening efforts with community providers and education systems through school-based vision programs. Finally, we discuss the impact of the COVID-19 pandemic on vision screenings and children’s access to vision care. In Part 2, we provide an overview of future directions and priority areas for pediatric vision screenings in the United States.

## 2. Part 1: Rationale and Guidelines for Pediatric Vision Screenings

### 2.1. Why Do We Conduct Pediatric Vision Screenings?

A pediatric vision screening is an evaluation to detect reduced visual acuity and/or risk factors that threaten the growth and development of the eye and visual system in children [4]. As many eye problems may be asymptomatic, vision screening plays an important role in detecting visual disorders in children without signs of disease or impairment, facilitating early diagnosis and treatment, and decreasing the impact that any untreated condition may have on visual development, as well as the educational and social progress of these children [5]. While this article focuses primarily on the pre-school and school-age population, resources for vision assessments in newborn and young children are also available [6].

According to criteria defined by the World Health Organization, vision screening is an example of an effective screening program as it aims to detect an important health problem (i.e., irreversible, vision-threatening with potential lifelong impact on an individual’s quality of life) for which there is an accepted treatment (i.e., conventional standards of clinical care as endorsed by major professional medical organizations) and a suitable screening test to diagnose those at risk for the condition [7]. Vision screenings may be conducted in educational-, community-, and public health-based settings, or in the medical home using recommended tools and procedures appropriate for the venue. Furthermore, screening is a cost-effective method to identify children in need of evaluation by an eye care professional [8,9].

The goals of vision screening and the vision conditions for which a child may be at risk can differ by age. In infants and young children, one of the most important causes of vision impairment is amblyopia, a disorder of visual development secondary to abnormal visual stimulation that can lead to permanent vision loss in the affected eye [10]. Risk factors associated with the development of amblyopia include strabismus (ocular misalignment), vision deprivation caused by obstruction of the visual axis (e.g., cataracts or ptosis), and significant uncorrected refractive errors (e.g., myopia, hyperopia, astigmatism, and anisometropia) [10]. In recent population-based studies on United States children less than 6 years of age, the prevalence of amblyopia and its risk factors has been estimated to be around 1–6% [11,12]. With early identification and prompt treatment, both the prevalence and severity of amblyopia could be significantly reduced [13].

For school-aged children 6 years and older, in addition to the monitoring of amblyopia risk factors until approximately the age of 9, the goals of vision screening expand to include the detection of risks for uncorrected refractive errors and other eye conditions (e.g., strabismus, ptosis, cataract) that may emerge for the first time throughout childhood and potentially impact a student’s academic and social functions [14]. Though the incidence of refractive error varies significantly by age, race/ethnicity, and other regional/geographical factors, the prevalence of refractive error has been increasing, albeit not uniformly, worldwide in recent decades [15]. It has been reported that 25% of children between the ages of 6 and 18 years need corrective lenses to address refractive errors [16]. In the school-age population, correction of refractive errors through the provision of eyeglasses has been shown to have a positive impact on students’ academic performance [1].

### 2.2. Professional Guidelines for Vision Screenings

In the United States, government, advocacy, and service organizations have developed policies on vision screening, including the American Academy of Ophthalmology (AAO), American Association for Pediatric Ophthalmology and Strabismus (AAPOS), American Academy of Pediatrics (AAP), American Association of Certified Orthoptists (AACO), and United States Preventive Services Task Force (USPSTF) (Table 1) [4,6,9,17,18,19]. Pediatric vision screenings are conducted worldwide, although with marked variation in how screenings are conducted [20]. It is generally agreed that vision screening should be performed throughout childhood, with suggested intervals varying by guidelines [4]. Furthermore, the sensitivity of multiple vision screenings is greater than that of one screening [4]. This is particularly true if different screening methodologies are implemented based on a child’s increasing age and abilities over time [4]. The ideal timing and components of pediatric vision screening, however, remain a matter of debate. As a result, pediatric vision screening guidelines differ slightly in the recommended age to begin screening, the intervals at which screening should occur, and the specific tests to be performed during screening. As current pediatric vision screening guidelines and recommendations have primarily focused on detecting amblyopia and its risk factors in the preschool age (3 to 5 years), before the critical period of visual development, there is an even greater lack of evidence examining the ideal timing and screening procedures to use in the school-age (6 years and older) population [14].

### 2.3. State Vision Screening Mandates

In addition to the variation in screening guidelines across professional organizations, pediatric vision screening requirements also vary significantly across states. According to a 2021 analysis of state vision screening requirements in the United States, approximately half (*n* = 26) of US states require vision screening for preschool-aged children, while the majority of states (*n* = 41) require vision screening for school-aged children [22,23]. Across states, there is also marked variation in the mandated screening methods, screening criteria, setting in which screenings occur, age/grade levels screened, and reporting requirements [23]. The lack of standardization and wide variation in state regulations point to a need for the development of evidence-based criteria for vision screening programs, especially for school-aged children.

### 2.4. Who Conducts Vision Screenings?

Personnel who conduct vision screenings frequently vary depending on the setting in which a screening occurs. In the outpatient pediatric setting, nurses or other clinical staff often perform vision screenings as part of well-child checks. Pediatricians and other physicians who work with pediatric patient populations are also trained to identify common and/or serious pediatric ocular pathology, especially those that frequently warrant management by pediatric ophthalmologists [24]. In the community setting, vision screenings may take place at schools, preschools, daycares, or at community-wide events such as health fairs. These community vision screenings are typically carried out by trained lay personnel or other health professionals, including nurses [4].

Nurses, other health care professionals, and lay screeners who perform vision screenings should be trained to elicit specific risk factors for vision problems, detect structural eye problems, and/or assess visual abilities or acuities in every age group [4]. They should also be trained in the techniques used to test younger children and those with neuropsychological conditions or developmental delays [4].

### 2.5. Vision Screening Components

The components of vision screening depend upon a child’s age and level of cooperation [4]. Vision screening protocols also vary depending on the setting in which screening occurs, the state and federal mandates for vision screening in the pre-school and school-age population, the availability of instrument-based screening equipment, and the screener’s level of training.

#### 2.5.1. Visual Acuity Testing

Traditional subjective visual acuity testing remains the primary approach for vision screening [4] in school-age children, especially in community- or school-based settings where large-scale screening often occurs [25]. Distance visual acuity is often tested by having the child name objects or letters on a wall-mounted or computer displayed eye chart with age-appropriate optotypes. It is typically performed at a standard distance (10–20 ft), monocularly (testing one eye at a time), covering the other eye with a handheld occluder or adhesive patch/tape, and with the child wearing any necessary corrective lenses [25]. Some children as young as 3 years old may be able to participate in subjective distance visual acuity testing, though accurate testing is more often achieved with a higher success rate in children 4 years and older and becomes more reliable and efficient in those aged 6 years and older [19,26]. Children who are unable to complete subjective visual acuity testing are considered untestable by that approach and are typically rescreened within 6 months or referred for a comprehensive eye examination [25]. It is important to understand which children are testable and untestable by the screening techniques employed, especially as untestable children have been reported to have a higher prevalence of vision issues and may benefit from closer monitoring to ensure access to follow-up care [27].

#### 2.5.2. Ocular Alignment and Stereoacuity Testing

Ocular alignment and stereoacuity testing are conducted to determine whether a child’s eyes are aligned and working together to see things in three dimensions. The development of strabismus in children may occur at any age; although often an isolated finding, it may also represent serious orbital, intraocular, or intracranial disease [26]. Strabismus is also a frequent cause of amblyopia in younger children. Examination techniques include the cover test and the corneal light reflex (Hirschberg) test, which are helpful for the detection of strabismus and for the differentiation of true strabismus from pseudostrabismus (an optical illusion created by prominent epicanthal folds that makes eyes appear misaligned). When the eyes are aligned and working together, the brain is able to blend the separate images from each eye into one image, which allows for perception of a three-dimensional space, or stereopsis. Common tests for stereopsis include the Preschool Assessment of Stereopsis with a Smile (PASS) II test and the Random Dot E (RDE) test of stereoacuity [14].

#### 2.5.3. Color Vision Testing

Pseudoisochromatic plates with numbers or shapes can be used to detect color vision abnormalities. Currently, 11 states require color vision screening in the school-age population with significant variation in screening protocol [23]. It is estimated that 8% of Caucasian males and 0.4% of females have color vision deficits [28]. Diagnosing color vision deficiency in children who are not experiencing any visual issues may be of little clinical value, as there is limited evidence regarding the negative impact of color vision deficits [14]. Therefore, color vision testing is usually not routinely recommended for mass school-aged vision screening, unless required by the state [14]. Identification of color vision deficits may, however, be of importance to parents or teachers for making accommodations at home and in the classroom.

#### 2.5.4. Instrument-Based Vision Screening

Instrument-based screening refers to vision screening using automated technology, such as photoscreening and/or autorefraction devices. These devices are usually handheld, easy to operate, and require minimal cooperation from the child, making them a useful alternative to subjective visual acuity testing for children who are pre-literate or who have developmental delays [26].

Photoscreening instruments use an off-axis image and assessment of the eyes’ red reflex to identify optical characteristics of the eyes and estimate refractive error, media clarity, ocular alignment, and eyelid position [4,26]. Photoscreeners assess both eyes simultaneously; the images generated can be interpreted by trained operators, by a central reading center, or with computer software [26]. Autorefractors, in contrast, use optical methods to estimate the refractive error of each eye separately, and as such, are limited in their ability to detect ocular misalignment. Autorefractors do, however, remain useful in detecting anisometropia (unequal refractive error), which is the most common cause of amblyopia among children whose amblyopia is not detected at an early age [26].

Instrument-based vision screening techniques have demonstrated high sensitivity and specificity for the detection of amblyopia risk factors in preschool-age children and offer the ability to adjust referral criteria to meet the desired sensitivity and specificity levels [4]. However, little evidence currently exists in the school-aged population regarding the sensitivity and specificity of instrument-based screening, especially in comparison to traditional optotype-based visual acuity screening [14]. There are also different equipment portability considerations, costs, and staffing needs for screenings that utilize instrument-based approaches. In addition, no national United States guidelines are available for instrument referral criteria settings for refractive error cutoffs specifically for the school-age population. Therefore, whether instrument-based screening alone should be recommended for mass screenings of school-aged children remains an area in need of further research [4,14].

### 2.6. Persistent Unmet Need for Vision Screenings in the United States

In recent years, the rates of vision screening in the United States have progressively decreased. The National Survey of Children’s Health (NSCH) showed that caregiver-reported vision screening declined by 13.6% from 2016 to 2020 (9.6% to 60.1%) [29]. This downward trend continued into 2021 as the most recent NSCH data suggest slightly fewer than 55% of children under 17 years completed vision screening [30]. Furthermore, screening rates vary significantly across different socioeconomic and demographic groups [29,30]. Children without insurance or who have gaps in insurance coverage are less likely to receive vision screening and are more likely to report higher rates of unmet vision needs [31].

Across race and ethnicity, Black and Hispanic children present to vision screenings with higher levels of visual impairment and less prior eye care, respectively [32]. Other groups disproportionately affected by low rates of vision screening include those with limited access to preventive care or well-child checkups and children living in families living at 200% below the federal poverty line [30,33].

Vision screening also varies across geographic location in the United States. Caregivers of children aged 3 to 5 years living in states without vision screening mandates are less likely to report that their child had their vision tested [33]. Following the identification of non-refractive vision issues, there is variation in reports of persistent visual difficulties across demographic groups, with non-Hispanic black females reporting a particularly high rate of persistent vision problems (89%) [34]. Public policy changes and a greater understanding of social determinants of health are needed to increase the rates of vision screening and address the inequalities in access to preventive vision care.

### 2.7. Referral for Eye Care following a Vision Screening

Available research shows that children frequently experience difficulty accessing follow-up after referral from a vision screening. In Pennsylvania, a state that requires annual vision screenings for all school-age children, over half of the children who meet referral criteria during screenings do not receive follow-up services [35]. Barriers to care may exacerbate children’s difficulties in accessing follow-up from vision screening referrals. In a study across Michigan and Maryland, children with Medicaid insurance had decreased odds of successfully scheduling follow-up appointments when referred after a vision screening as compared to children with private insurance [36]. When a child’s vision screening results meet referral criteria, follow-up is indicated. Children should be connected with a licensed ophthalmologist or optometrist to receive an eye exam. Depending on the results of the exam, children may be prescribed eyeglasses or receive ongoing care for more complex eye conditions.

School-based vision programs (SBVPs) are one approach to increase rates of follow-up from vision screenings. SBVPs typically involve partnerships between community organizations and schools to provide follow-up eye exams for students referred after vision screening. In addition, many SBVPs provide eyeglasses to children who need them. SBVPs typically require no out-of-pocket costs to students. For children with more complex eye conditions, SBVPs work to connect these students with local eye care providers for further evaluation and treatment. In the United States, SBVPs are operating in over 20 states and are continuing to grow in number [37]. SBVP programs have been shown to directly benefit students’ academic performance [37]. Notably, the academic benefits of SBVPs may be most pronounced for previously low-achieving students and students enrolled in special education classes, including children with learning disabilities or language impairments [37]. Thus, by partnering with schools and local eye health stakeholders to facilitate follow-up care, SBVPs offer a useful extension to traditional vision screenings. School-based vision programs also align with the principles of a whole child approach, as outlined in the WSCC model [3], recognizing the inter-relatedness of vision and eye health to a child’s success in the classroom. The accessible, school-based setting and additional supports they provide may allow SBVPs to have an even greater impact for children living in areas with existing barriers to care, such as high-poverty urban environments.

### 2.8. Vision Screenings during the Pandemic

The COVID-19 pandemic resulted in substantial disruptions in school operations, including short- and long-term school closures beginning in March 2020 as part of the COVID-19 transmission mitigation measures. These closures negatively impacted the delivery of various school-based health services, including school-based vision screenings. During the 2020–2021 academic year, over half of the states with vision screening mandates for school-age children either waived or modified their existing vision screening requirements, such as the grades screened or assessments included, while only approximately 30% of states continued screening without modification [38,39]. It is estimated that over 3 million students across the United States missed vision screenings during the 2020–2021 academic year as a result of the pandemic [39]. In addition, according to the NSCH data, there was an 85.7% relative increase in the number of children who did not receive vision care in 2020, as reported by their caregivers at the onset of the pandemic [29].

While necessary at the time as part of broad public health strategies, these disruptions in vision screening requirements may have long-term implications for children’s eye care, especially given the widespread use of virtual learning, increased screen time, and decreased time spent outdoors during the pandemic [40]. These factors, paired with the significant reduction in access to eye care services during the pandemic, may be playing a role in the increased prevalence of myopia reported in other countries [40,41,42,43]. In children aged 6 to 8 years living in China, the prevalence of myopia increased 1.4 to 3 times in 2020 compared to previous years [40]. Continued reporting will allow for a better understanding of the impact of the pandemic on children’s vision care.

## 3. Part 2: Future Directions and Priority Areas for Vision Screenings

Pediatric vision screening is important to identify children at risk for uncorrected refractive error or other eye conditions. Based on our experience, we believe there are many opportunities to improve on our current approaches to vision screening. First, given the wide variation in screening practices, especially in the school-age population, there is a need for standardized guidelines across states. Standardized guidelines may build on recommendations of professional organizations to aid in detecting early eye disease while also understanding the interrelatedness of vision and academic performance for older, school-age children [13]. As more information becomes available regarding the optimal components and frequency for pre-school and school-age vision screenings, standardized guidelines would continue to evolve in response to available research.

There are also opportunities to use technology to improve the vision screening processes. Current data storage and data entry practices for vision screening information are frequently difficult to access for key stakeholders, such as parents, caregivers, and pediatric primary care providers. It is also often difficult to identify whether a child has received a prior vision screening or to track vision screening results over time. The creation of state-wide portals to house standardized vision screening information would address many of these issues; standardized portals would increase accessibility to data, reduce redundant testing, and aid the identification of children in particular need of vision screenings or follow-up after a vision screening referral. In addition, new technological advances in vision screening equipment may have a role in future vision screenings. Instrument-based devices using technology based on visual evoked potentials, retinal birefringence, and eye-tracking technologies are currently in development and may provide additional means to assess visual acuity and ocular health in young children [26,44]. Smartphone applications can expand access to vision screenings, especially in areas with limited access to traditional vision screening programs [45,46].

Connecting children to eye care following a referral continues to be a challenge and should remain a top priority in developing systems to address barriers to pediatric eye care. Building partnerships and communication with vision health stakeholders are also key priority areas. Partnerships with local eye care providers may help vision screening programs to better navigate follow-up after vision screenings and coordinate care for children with complex ocular conditions. Eye care providers may also inform and support efforts to engage with the local community. Parents’ and children’s awareness of eye health and vision screening programs can affect participation in screening programs in the short-term [37] and may have numerous long-term benefits for vision health. In addition, both local eye care providers and community members are valuable sources of feedback for vision screening entities to tailor their practices to the current needs of their local areas.

The COVID-19 pandemic has also broadly affected children’s access to care, including children’s access to vision screening. As communities continue to recover from the ongoing impacts of COVID-19, there may be an increased need for vision screening services, especially for children who may have missed routine services during the pandemic. Future public health efforts may aim to better understand and address the pandemic-related disruptions in pediatric vision screenings and eye care [38].

## Figures and Tables

**Table 1:** Summary of Professional Organization Vision Screening Guidelines/Recommendations.

Professional Organization	Year Published/Updated	Setting/Screeners	Screening Interval	Assessment
American Academy of Ophthalmology (AAO) [4]	2018	Primary care or community setting/Physicians, nurses, other health care providers, and lay individuals specifically trained to perform vision screening	Age 3, 4, 5, and every 1–2 years after age 5	Red reflex, external inspection, pupil examination, corneal light reflection, instrument-based screening (subjective visual acuity testing preferred over instrument-based screening in children able to reliably participate), distance VA (monocular; LEA Symbols, HOTV, and Sloan Letters are preferred optotypes)
American Association for Pediatric Ophthalmology and Strabismus (AAPOS) [17]	2019	Primary care setting or community setting (daycare programs, churches, schools, and health departments)/Pediatricians, family practitioners, nurses, and technicians.	Repeat every 1–2 years after age 5	Ocular history, external inspection of eyes and lids, ocular motility assessment, pupil examination, red reflex examination, vision/visual acuity assessment (Sloan preferred over Snellen), Ophthalmoscopy
American Academy of Pediatrics (AAP) [19]	2016	Primary care setting/Pediatricians or primary care physicians	Begin at age 3, then at age 4, 5, 6 and 8, 10, 12, and 15 [21]	Ocular history, external inspection of lids and eyes, red reflex, pupil examination, ocular motility assessment (cross cover test), ophthalmoscopy (if possible), distance visual acuity w/ age-appropriate optotype (Sloan, Snellen, HOTV, LEA), instrument-based (if unable to use age-appropriate optotype); one-time color vision for boys
American Association of Certified Orthoptists (AACO) [18]	2021	--	Vision screening every 1–2 years beyond 5 years of age	Ocular history, subjective visual acuity (monocularly), instrument-based vision screening (if subjective visual acuity testing not possible), cover-uncover test, corneal light reflex, red reflex, inspection of eyes and lids
United States Preventive Services Task Force (USPSTF) [6]	2017	Primary care setting	Vision screening at least once in all children aged 3 to 5 years to detect amblyopia or its risk factors	Visual acuity (picture identification-LEA, HOTV, Snellen, Tumbling E), Stereoacuity (contour stereotest, moving dynamic random dot stereosize test), ocular alignment test (Hirschberg, cross cover test, Bruckner test), instrument-based screening (photoscreening/autorefraction)

## Data Availability

Not applicable.
